# A Report on ASEMV2020

**DOI:** 10.20517/evcna.2020.08

**Published:** 2020-12-30

**Authors:** Michael W. Graner

**Affiliations:** Department of Neurosurgery, University of Colorado Anschutz Medical Campus, Aurora, CO, 80045, USA.

**American Society for Exosomes and Microvesicles (ASEMV**) held its Annual Meeting, ASEMV2020 on November 16-19, 2020. The precursor meetings of Exosomes & Microvesicles 2011 and 2012 (in Orlando, FL, USA) were among the earliest exosome/extracellular vesicle meetings held in the United States, with many international attendees. The Society coalesced under the ASEMV banner in Orlando in 2013, and has held Annual Meetings ever since. This year’s meeting was obviously substantially different, conducted virtually due to the ongoing COVID-19 pandemic. This changed much of the usual meeting dynamic, but the conference maintained its high level of scientific presentations and discussions that are the heart and soul of the Society and its yearly meetings.

Previous ASEMV Meeting sessions consisted of talks with mixed topics that allowed for a broad presentation of information that attendees might have surprisingly found more interesting than they may have ordinarily anticipated. For ASEMV2020, however, the Organizing Committee chose to group talks by topics, sometimes covering two sessions. The rationale was to have all questions pertaining to the entire session covered in one panel discussion period, after all talks for that topic were completed. All speakers were present in the roundtable forum. The audience members entered their questions via a chat function that was enabled during the talks and the discussion. Session moderators compiled questions and directed them to each of the session’s speakers accordingly. Attendees could also email questions to speakers, or engage by messaging the community forum. These processes enabled some semblance of the lively scientific dialogue, which is a hallmark of ASEMV Meetings.

ASEMV2020 was hosted online by Designing Events. The Meeting had 181 registrants from 14 countries and 3 sponsors (NanoView Biosciences [gold]; Izon Science [silver]; and Spectradyne Particle Analysis [bronze]). ASEMV2020 spanned four days, comprising three to four sessions and two related discussion periods per day, plus one poster session. There were also two ASEMV Working Group (WG) Meetings, including the Resource Sharing WG and the newly formed Diet and Nutrition WG. The Meeting was flanked by grants workshops, and ended with an Awards Ceremony with prizes for Young Investigator talks and poster presentations.

ASEMV2020 opened with remarks from ASEMV President, Steve Gould (Johns Hopkins University, US) and ASEMV2020 Organizing Committee Chair, Louise Laurent (University of California San Diego, US), welcoming attendees and describing the new format for this year.

Next came the first of the grants workshops organized by Fatah Kashanchi (George Washington University, US). This featured Program Directors from the US National Institutes of Health (NIH) whose Centers and Institutes provided funding on exosome-related projects.

The grants workshop opened with a presentation from Matthew Young (National Cancer Institute, (NCI), Cancer Biomarkers Research Group, Division of Cancer Prevention) who focused on the utility of extracellular vesicles and particles (EVPs) in cancer detection via liquid biopsy. He covered “hopes and hypes” in the area, noting that as of yet, there are no US Food and Drug Administration (FDA) approved clinical uses for exosomes/extracellular vesicles, although trials for both biomarker and therapeutic applications are underway. He emphasized upon the hope that EVPs could be used for plus/minus cancer detection (cancer *vs.* non-cancer), across various cancer types and stages. This would require rigor in EVP separation methods and biomarker identification, with applicability at either standard clinical lab facilities or in specialized centers. He advertised PAR-20-253, an NCI Funding Opportunity Announcement for innovative research in vesicle separation and characterization, the goal of the PAR is to promote rigor and reproducibility in field.

Matthew was followed by John Satterlee from the National Institute on Drug Abuse (NIDA), Division of Neuroscience and Behavior, who presented a broad overview of the NIH budget and funding breakdown, then narrowing down to NIDA. In the background of an increase in drug overdose deaths in the US in 2019, he referred to several existing exosome/EV funding opportunities and currently funded projects on neuroscience and neuropathology, associated infectious diseases, and diagnostics where there is overlap with the Extracellular RNA Communications program (see below). John encouraged everyone with an interest in NIH funding opportunities to subscribe to the “NIH Guide to Grants and Contracts” (https://grants.nih.gov/grants/guide/listserv_dev.htm); to contact Program Officers to learn more about particular funding areas; and to study the “ten commandments for preparing a compelling R01 application” that was presented by Fatah Kashanchi in a grants workshop session on the final day of the Meeting.

The session closed with a presentation from Christine Happel (National Center for Advancing Translational Science (NCATS) who is Scientific Program Director of the NIH Common Fund’s Extracellular RNA Communications Program (ExRNA) that sponsored the Extracellular RNA Communications Consortium (ERCC). She reported the accomplishments of ERCC’s Phase 1, including the massive accumulations of biofluid samples and associated exRNA studies and profiles; analyses of technologies and methodologies for extracellular vesicles (EVs) separation and exRNA identification; and the creation of data analyses and coordination protocols for information processing. ERCC developed a portal for access to these data, protocols, and other resources (e.g., the exRNA Atlas), to be found at exRNA.org (https://exrna.org/). Christine touted a number of landmark papers from the ERCC published in 2019 (Cell journals) that culminated from those data. ERCC Phase 2 will continue research in these areas, with interests in EV and exRNA heterogeneity; improvements in isolation, characterization, identification, and data processing of EVs and exRNAs; and an increased interest in single exosome/EV isolation and cargo determination. The latter, in particular, relates well to an advertised RADx (Rapid Acceleration of Diagnostics) funding announcement, RFA-OD-20-018, seeking approaches to detect, test, validate, and implement testing for SARS-CoV-2 in the COVID-19 pandemic. She ended with a list of exosome/EV-related funding announcements across NIH Institutes, and commented on the future of vesicles and exRNA in terms of basic biology and translational applications.

The next 2 sessions focused on exosome/EV cargo loading and cargo unloading. Session Chairs were Juan Pablo Tosar (Universidad de la República, Uruguay) and Andrew Leidal (University of California San Francisco, US). Alissa Weaver (Vanderbilt University, US) addressed a controversy in the EV field: is EV-associated Argonaute an artifact or is it selected cargo? She noted that extracellular RNA in blood is prevalent in non-vesicular forms, likely released from necrotic cells, and largely held by RNA-binding proteins (RBPs), including Argonaute 2 (AGO2). Vesicular RNA in blood is less abundant, but is a product of active secretion. These forms may represent different functional capacities, as well. She noted that different cell status (mutations, signaling) result in different RNAs and RBPs that are expressed and localized in cells, and differentially segregated into vesicles. She provided compelling evidence that serum (such as fetal bovine serum) is a source of non-vesicular AGO2 and microRNAs, which further complicates the assessments of AGO2 and miRNAs from cells grown in serum-containing media. Thus, the cell state, mutational and signaling status, and growth conditions all contribute to AGO2 and likely, other RBPs, and their bound RNAs in terms of vesicular or non-vesicular entities.

Leonid Margolis (NIH, National Institute of Child Health and Human Development, US) followed next with a presentation on EV-associated cytokines and their implications for cell-cell communications. This topic highlights broad questions of how exosomes and other EVs are addressed to particular cells, and how the EV cargo affects recipient cells. EVs may play roles in protecting contents and applying appropriate ‘shipping addresses’. Leonid pointed out that cytokines are encapsulated within EVs, and are also present on EV surfaces. These operate as systems, and that the cytokine surface *vs.* lumenal localizations are not independent, but reflect changes in parental cell states. Differing soluble *vs.* cytokine-EV states are evident in blood of patients experiencing post-radiation fatigue syndrome and in a form of myocardial infarction, among other pathologic settings. EV-associated cytokines appear functional, and have an impact on recipient cells due to their selective delivery onto cells with appropriate receptors. Improved understanding of cytokine loading and targeting of such EVs could enlighten our concepts of cell-cell communication.

RNAs of various types are described as exosome/EV cargo, begging questions such as: how the RNAs are loaded, are they transferred to recipient cells, and are the RNAs functional in the recipient cells. Olivier de Jong (University Medical Center Utrecht, The Netherlands) reported studies in this area that employed an innovative CRISPR-Cas9 system, which they called CRISPR operated stoplight system for functional intercellular RNA exchange (CROSS-FIRE). It utilizes transfer of single guide RNAs (sgRNAs) that are capable of driving reporter signals in recipient cells in a non-contact system, thus indicating the loading of sgRNA into EVs in donor cells, and functional transfer to recipient cells measured by fluorescent readout. This transfer worked with various donor and recipient cell types. The system uncovered genes involved in endocytosis, extracellular matrix adhesion, and intracellular membrane trafficking that affected RNA transfer via EVs (ITGB1, ROCK1, RAB5, RAB7). For translational development, the system appears more efficient at functional RNA transfer than with lipid nanoparticle-based products, suggesting therapeutic uses of mRNA or siRNA transfer.

In a display of EV cargo (or lack thereof) relevant to disease processes, Laura Ferraiuolo (University of Sheffield, UK) introduced the neurodegenerative disease amyloid lateral sclerosis (ALS). The most frequent mutation in the familial version of ALS (35%) and spontaneous ALS (11%) is in C9ORF72, which can lead to several potential (and overlapping) pathological situations. One potential mechanism is that the C9ORF72 translation product may inappropriately sequester RNAs, thus altering cell function and cell crosstalk. Laura showed that transfer of EVs from astrocytes, generated from patients with amyloid ALS, to healthy neurons induced neuronal cell death. Scores of small RNA levels were significantly altered in those EVs, including downregulation of miR494, which affects pathways controlling axonal growth cone collapse. Supplementation of miR494 into astrocyte EVs and application of the EVs onto neurons rescue neuron survival, neuronal length, and complexity (increases in nodes and intersections). These studies reveal the potential of therapeutic cargo for use in EV transfer, based on knowledge of the EV-driven pathology.

Crislyn D’Souza-Schorey (University of Notre Dame, US) spoke of microvesicles (MVs), which are extracellular vesicles that bud from cell surfaces, and the loading of pre-miRNA cargo into tumor EVs. She noted the extraordinary heterogeneity of shed vesicles, particularly those released by tumor cells. Her group has identified ARF6, which is involved in endosomal/endocytic recycling; ARF6 activation correlates with increased miRs in MVs. They also found that XPO5 is a binding partner of ARF6; XPO5 plays roles in pre-miR transport from the cell nucleus to the cytoplasm, and is found in tumor MVs, which contains a pre-miR processing machinery. Tumor MVs contain complexes of ARF6/XPO5 and pre-miRs; translocation of XPO5 to the MVs involve a complicated interaction involving CK2, RAN and cytohesion family guanine exchange factors. These routes suggest that the intricate mechanisms for the delivery of cellular cargo into different EV types further display the complexity of these biologic and pathologic processes.

Qin Zhang (Vanderbilt University) finished out the session discussing a recently identified form of extracellular particle, the exomere. Exomeres are non-membranous particles (~35 nm diameter) of unknown biogenesis and no clear biologic function. Researchers previously used a biophysical separation technique called asymmetric-flow field-flow fractionation (AF4) to isolate and identify exomeres; Qin performed high speed ultracentrifugation (UC) on cell culture medium (167K × g; collect supernatant; UC again @ 167K × g; harvest pellet) to obtain similar extracellular particles, which were proteomically distinct from small EVs/exosomes. Exomere-enriched proteins included glycolytic enzymes and AGO RNA binding proteins. Exomeres also contained the EGFR ligand, AREG, which in that format was capable of inducing prolonged EGFR signaling in recipient cells, leading to enhanced tumor organoid growth. Another exomere-enriched protein is the sialyltransferase, STGAL1 (membrane and soluble forms), which is highly enzymatically active in exomere form on recipient cells. Curiously, the SARS-CoV-2 spike protein receptor, ACE2, is displayed on exomeres from some cell lines and can bind the viral spike protein via the S1 receptor binding domain, suggesting that exomeres could serve as decoys against SARS-CoV-2 spike protein-initiated infection.

The final session of the first day of ASEMV2020 was devoted to analytical techniques, and was hosted by Kendal van Keuren-Jensen (Translational Genomics Research Institute, US) and Tijana Jovanovic-Talisman (City of Hope, US). The first speaker was Lisa Meyer (Exosome Diagnostics, US; Germany) who presented an exosome/EV separation and characterization workflow using products from several partner companies. This included a kit-based separation technology, an automated Western blot device in the form of a capillary flow electrophoresis apparatus, and validated primary antibodies against exosome/EV and theoretical non-EV proteins to determine potential contaminants. She showed results confirming the detectability of appropriate target proteins in the correct (EV-containing) fractions from the separation scheme. The system provides scalability, rapid quantification, and good detection limits. They are expanding the antibody repertoire and improving the separation modules, going forward.

Ryan McNamara (University of North Carolina, Chapel Hill, US) introduced a super-resolution microscopy technique called direct stochastic optical reconstruction microscopy (dSTORM). As exosome/EV sizes are below the limits of resolution of conventional light microscopy, more advanced forms of imaging are necessary to accurately discern single EVs and their characteristics, thereby avoiding non-physiologic manipulations that are likely pursued in what might be done for electron microscopy. dSTORM has multi-channel capability that could detect two different membranes dyes within the lens apochromatic correction limit (< 20 nm), i.e., essentially co-localized. When using labeled tetraspanins, they determined that there is an offset in the overlay of the membrane protein and the membrane dye, which they hypothesized to be the localization of the tetraspanins in lipid raft microdomains. The biophysical characteristics shown were consistent with those found from other methodologies (e.g., nanoparticle tracking analysis, resistive pules sensing, and surface plasmon resonance).

Frederik Verweij (INSERM, France) rounded out the session with a talk on *in vitro* and *in vivo* exosome tracking. He described a novel live cell imaging technique using pH sensitive reporters visualized by total internal reflection fluorescence (TIRF) microscopy. With tagged tetraspanins (CD63, CD81) or other endosomal markers, they could visualize which endosomal sub-compartments fuse with the plasma membrane to release exosomes. They have extended these studies to *in vivo* settings using zebrafish models. The remarkable resolution of their technology allows tracking of release of exosomes, as well as recipient cell uptake throughout the organism; e.g., macrophage and endothelial cell scavenging, and internalizing exosomes released by cells of the yolk syncytial layer. Following the fate of such internalized exosomes, it seems that many exosomes are degraded in the endo-lysosomal system. Further, inhibiting the biogenesis of exosomes in zebrafish embryo caudal vein plexus (CVP) led to reduced growth of that tissue, implying a role for exosomes in trophic support of the CVP.

**ASEMV2020 Day 2** began with an EV Function session, chaired by Michael Graner (University of Colorado Anschutz, US) and Leonid Margolis (NIH, US). It started with a joint presentation by Jay Debnath and Andrew Leidal (University of California San Francisco, US) on secretory autophagy and EVs. Jay introduced the biologic processes of autophagy, particularly secretory autophagy, and their relationships to EVs and EV release. Given that knockdowns/knockouts of autophagy components had pleiotropic effects, Drew had developed a technique called proximity-specific biotinylation to identify new targets of autophagy-dependent secretion. This allowed for proteomic identification of > 200 such targets, many of which were exosomal/EV components and RNA-binding proteins, suggesting an intersection between autophagy and exosome protein-related secretion. Additional work showed the dependency of autophagy machinery on secretion of diverse RBPs as well as certain small RNAs (including snoRNAs) in small EVs/exosomes. They believe this represents a sub-routine of the autophagy pathway involving component (LC3) processing and lipidation, “LC3 dependent EV loading and secretion” (LDELS). Drew inhibited lysosomal activity (baflomycin, chloroquine), which revealed a re-direction of endo-lysosomal cargo released as EVs outside the cell in an autophagy-dependent manner, with autophagy proteins, including cargo receptors, prominent in those vesicles. Further genetic studies indicated that inhibition of autophagosome-lysosome fusion enhances cargo receptor release in EVs, in contrast to the requirements for LDELS.

Sarah Andres (Oregon Health and Science University, US) continued the autophagy theme, this time focusing on IGF2PB1/IMP1, an RNA-binding protein whose expression peaks during embryonic development in mammals, and decreases as the organism matures. In the intestine, damage causes upregulation of IGF2PB1/IMP1; this is also seen in GI cancers, where it acts in tumor progression and metastasis. The hypothesis was that IMP1 plays a role in endosomal and autophagic pathways (secretion *vs.* degradation) in colon cancers. IMP1 overexpression increased EV release, and modulated endosomal and autophagy pathways at least in some colon cancer cell lines. Using an enteroid (organoid) culture system (from crypt regions of intestinal epithelium), IMP1 overexpression in these non-transformed cells did not alter EV production, but had some impact on cargo, such as TSG101; these results are an on-going work. Thus, IMP1’s influence on EV biology via endosomal and autophagy pathways appears to be cell-type and context dependent.

Inge Zuhorn (University of Groningen, The Netherlands) presented next on exosomal cargo fate upon uptake by recipient cells. Their work originated from challenges that accompanied nanoparticle-based drug delivery to the brain; subsequently leading to EV-based delivery into the brain with functional release of cargo into target cells. Inge presented four hypothetical exosome cargo release scenarios (direct fusion to plasma membrane; “kiss-and-run” fusion at the ER; endosomal membrane fusion - aka, back fusion; and endosomal rupture). They used an elegant system to dissect these mechanisms, starting with a cell line expressing a GAL3 fluorescent reporter that is detectable upon binding beta-galactoside aggregates, which would be present if endosomes ruptured. Upon exosome incubation, such rupture was not evident. They next used exosomes from cells expressing intralumenal GFP-tagged CD63 that were loaded onto cells expressing a labeled (mCherry) anti-GFP nanobody intracellularly. If exosomes fuse at the plasma membrane, the red nanobody would accumulate there; if exosomes back fuse inside the endosome, the red nanobody would accumulate intracellularly. There would be co-localization with GFP at each of these sites. Their experiments revealed intracellular co-fluorescence, indicating endosomal release; and correlative light and electron microscopy (CLEM) also showed lysosomal localization, suggesting movement through the endo-lysosomal pathway. As these results suggested that protein transfer may occur via exosomes, they attempted treatment of a Huntington’s Disease (polyQ, HTT-Q74) murine model. They used their exosome loading protocol to place the HSP40 chaperone homolog DNAJB6 into exosomes. In intrathecally injected transgenic mice, they showed reduction in poly-Q aggregates in mouse brains and spinal cords, indicating functional transfer of the chaperone to reduce aggregates.

Continuing the theme of *in vitro*/*in vivo* work, Nicole Noren Hooten (NIH National Institute on Aging, US) introduced the potential role of EVs in type 2 diabetes mellitus (T2DM). Previous studies found that larger EVs (microparticles) derived from blood cells were elevated in patients with T2DM. Nicole’s work examined smaller vesicles (exosome and microvesicle range) in a large longitudinal study was comprised of euglycemic individuals, pre-diabetics, and T2DM patients (NIH HANDLS study). Precipitated plasma EV concentrations were elevated in T2DM patients, and this increased as pre-diabetic patients progressed to full disease. T2DM EVs had lower amounts of insulin signaling proteins and induced inflammatory responses when incubated with monocytes and B cells, thus suggesting that those EVs may promote inflammation in patients. Longitudinal and cross-sectional analysis of EV inflammatory proteins showed that levels of VEGFA were associated with diabetes development in this expansive cohort. They followed this with differential centrifugation of plasma EVs (10K × g and 120K × g fractions). Curiously, the 10K EV fraction from T2DM patients increased endothelial cell migration *vs.* those EVs from euglycemic controls, and those cells showed increased actin rearrangements and lamellipodia formation. This raises the possibility that plasma EVs in T2DM patients may contribute to detrimental vascular changes that are related to the diabetic pathology.

Y. Peng Loh (NIH, National Institute of Child Health and Human Development, US) completed the EV Function session with her group’s latest work on exosomal carboxypeptidase E (CPE) in tumor vesicles. CPE mRNA overexpression is correlated with poorer overall survival in patients with hepatocellular carcinoma (HCC), and is generally elevated in serum exosomes of cancer patients as well as in highly malignant cancer cell lines. *In vitro*, exosomes from high metastatic HCC (more malignant) cell lines induce proliferation and invasion in lower malignant HCC cells, and this can be blunted if the exosomes contain shRNA against CPE. Loh *et al.*, transfected CPE shRNA into HEK exosome producer cells to treat high metastatic HCC cells with those exosomes, which reduced proliferation in those cells, perhaps by suppressing CCND1 and MYC. This suggests that CPE is a good cancer biomarker, a good anti-cancer target, and that exosomal delivery of shRNA may be a therapeutic avenue against cancer.

With increasing interest in the roles of EVs in microbiology, the next session featured talks on bacterial EVs, hosted by Meta Kuehn (Duke University, US) and G Marcela Rodriguez (Rutgers University, US). Marcela started the session with a talk on EV (membrane vesicle, MV) production in mycobacteria. Mycobacteria MVs display phospho- and glycolipid content consistent with the plasma cell membrane, but the bacteria is surrounded by a thick cell wall, which makes the MV release somewhat of a mystery. Mycobacterial MV production is regulated by several genes; strains lacking the “vesiculogenesis and immune response regulator” (viR) gene demonstrate enhanced vesicle production. Environmental iron availability also regulates vesiculation; MV impacts’ on host cells frequently involve immune modulation associated with pathogenesis. Searching for common genes involved in conditional upregulation of MV production, they associated the IniBAC operon with increased vesiculation. This encodes dynamin-like proteins (DLPs) necessary for cargo loading and vesicle release, and thus may be targets for therapeutics.

Tatsuo Kurihara (Kyoto University, Japan) also discussed cargo loading into extracellular membrane vesicles (EMVs) from Shewanella vesiculosa strain HM13. The most abundant protein in this bacterium’s vesicles is called P49, encoded in a gene cluster that also produces components that are likely to be membrane associated transport systems. Gene disruption analyses of various cluster members led to cellular accumulation or non-vesicular release of P49, and gene deletion of P49 of course deleted the protein entirely. Some of the genes encode cell surface polysaccharides that are also on EMV surfaces,which appears to be required for P49 loading into EMVs. P49 likely binds to those EMV surface polysaccharides. Collectively, this system could be exploited to load foreign proteins into a putative delivery system.

Jean C Lee (Brigham and Women’s Hospital, Harvard Medical School, US) followed next with a talk concerning the impact of Staphylococcus aureus (SA) EVs on host macrophage inflammatory responses. SA causes a diverse array of infections due to their varieties of virulence factors, which may serve as cargos in EVs such as pore-forming toxins and small peptide toxins. Macrophages uptake SA EVs via dynamin-dependent mechanisms. SA EVs trigger inflammasome activation via toll-like receptor (TLR) triggering (essentially as a priming step that causes IL6 release), and toxin-mediated inflammasome-driven cleavage of pro-IL1B and pro-IL18 to mature secreted forms. These were dependent on NLRP3 and CASP1 inflammasome components. These outputs are due to the toxin cargo in the SA EVs, which do not affect the TLR-driven IL6 release. The TLR signaling/IL6 release is driven by SA EV surface lipoproteins. Curiously, CASP1 activity was also dependent on lipoproteins, suggesting thereby that lipoproteins modulate the toxin content of EVs, which was verified by mutagenesis. The lipoproteins also modulate EV biogenesis and EV biophysical characteristics. The EVs thus function to protect the cargo and mediate virulence potentially through unmitigated inflammasome activation.

In a very interesting presentation on potential bio-utility of bacterial outer membrane vesicles (OMVs), Allison Z Werner’s (National Renewable Energy Laboratory, US) particular project involves generation of green products from biology; in this case, it is the conversion of lignin (e.g., in corn stalks) to usable and sustainable solid products. Lignin is the second most common biopolymer on earth (after cellulose) that remains behind from industrial processes and might be used to develop rigid products rather than form large quantities of burned waste. The material’s heterogeneity makes its deconstruction and re-utilization problematic. Pseudomonas putida, KT2440, is a bacterium capable of lignin catabolism as a part of the “biological funneling”, which is necessary to obtain simpler molecules for further development. Lignin promotes *P. putida* extracellular secretion in the form of OMVs. The research group characterized and performed exoproteomics on these vesicles over time under different lignin conditions. These included aromatic catabolic enzymes in the beta-ketoadipate pathway. There are many directions for future work in this new area.

Simon R Carding (The Quadram Institute and University of East Anglia, UK) presented information on the roles of Bacteroides thetaiotaomicron(Bt)-derived OMV, in microbiota-host crosstalk in the gastrointestinal tract. Bt is prominent in microbiomes residing at the mucosal interface in the gut. Bt OMVs are abundant and remarkably stable and naturally produced in the GI tract, where they are taken up by gut epithelium by dynamin-mediated endocytosis and macropinocytosis. Intracellular trafficking puts OMVs at the ER, Golgi, endosomal compartments and the nuclear membrane, but can apparently transcytose the epithelial barrier by passing between cells. Bt OMVs biodistribute beyond the GI tract into the liver with lower quantities in kidneys and lung, much like other EVs and nanoparticles. Unlike pathogenic bacterial OMVs, Bt OMVs tend to promote anti-inflammatory response in intestinal mucosa, probably from regulatory resident dendritic cells. Bt OMVs can also cross mucosal barriers in the lung, suggesting that these OMVs could be used as mucosal delivery vehicles for vaccines.

Hannah McMillan (Duke University, US) discussed bacterial vesicles in inter-kingdom communications with plants. While relatively understudied in plants, such OMVs are known to contain virulence factors, protein secretion components, and plant cell wall-degrading enzymes. Plant responses to bacterial pathogens are at levels of reactive oxygen species, erection of physical barriers, and production of antimicrobial compounds. Bacteria retaliate with the means to block and deactivate these immune responses, leading to further immune escalation from the plant that can include systemic responses and programmed cell death. Hannah investigated the roles of OMVs in these interactions. Using Pseudomonas syringae (Pst, plant pathogen) and fluorescens (Pf, not pathogenic) as OMV sources, she “infected” plant leaves with OMVs. Pst OMVs induced ICS1 protein expression (asso’ ciated with salicylate production against the bacteria). Bacterial OMVs produced protective immunity, where plants challenged with bacteria resisted bacterial growth and colonization, even if the non-pathogenic OMVs were used as an initial stressor. Also, both Pst OMVs and OMVs from non-plant pathogens could protect against different pathogenic challenges. The nature of OMV-driven protection at the level of the OMV is currently unknown and is an area of further study.

Blanca Rodriguez (Duke University, US) finished the session by talking about the immunomodulatory impacts of RNA in Staphylococcus aureus extracellular MVs. The RNA content of MVs was originally controversial, but has recently become generally accepted. These are mostly small RNAs; their sorting into MVs can be affected by growth conditions and the RNAs can be transported into host cells. There are many questions in this new area, including, the physical association of RNA with MVs, how RNAs are transmitted to host cells, and do they modulate immunity? It is known that SA nucleic acids are immunomodulatory, driving type I IFN responses, but the mechanism for this SA nucleic acid release and transfer is unclear, with the hypothesis that MVs are involved. Blanca found that exogenous RNA does not bind SA MVs, but does seem to have endogenously associated surface RNA (there is still RNA within the MVs that is extraordinarily stable, even in the presence of proteases and nucleases). The MVs promoted IFNB expression in macrophages; this expression was only mildly reduced if they attempted nuclease degradation prior to incubation with macrophages. MV uptake is dynamin-dependent, as is IFNB induction, suggesting endosomal entrapment where there are nucleic acid sensors (TLRs) that are responsible for most of the IFNB response. Further questions include mechanisms for release of the RNA, which RNA sequences are immune modulatory, and if alternate uptake and trafficking modes are involved.

**ASEMV2020 Day 3** started with sessions on the pathology of EVs, moderated by Janusz Rak (McGill University, Canada) and Michael Graner (CU Anschutz, US). The first speaker was Aleks Milosavljevic (Baylor College of Medicine, US) who related the effects of glioma EVs and brain endothelial cells. Glioblastoma (GBM), the deadliest of brain tumors, is a vascular tumor, implying tumor-driven modification of brain endothelial cells; Aleks’ group and others asked if EV-mediated angiogenesis differed from growth factor-driven angiogenesis. Using human brain endothelial cells as targets, EVs from GBM8 (stem cell glioma line) enhanced vascularization patterns in the endothelial cells in ways phenotypically similar to growth factors themselves; but which differed considerably at a molecular level involving distinct pathways. They suspected that this was occurring at the post-transcriptome level, and therefore examined the endothelial cell fraction that was deconvoluted from TCGA data. The results showed intersection of *in vitro* and *in vivo* perturbations. Further analyses showed a dominant effect of growth factors over EVs in methylation/epigenetics changes. miR-9-5p emerged from the data as a candidate extracellular RNA mediating angiogenesis upon EV exposure to endothelial cells based on overlapping miR expression data from EVs of several sources and its plasma biomarker status. Additionally, among downregulated gene profiles were miR-9-5p targets, including SOX7, which could be responsible for proliferative effects. These results suggest that EV-mediated angiogenesis, which has different features from growth factor-driven angiogenesis, may help explain the failure of anti-VEGFA targeting across several clinical trials in GBM.

The theme of detrimental impacts of disease-state EVs on normal cells was continued by Romano Regazzi (University of Lausanne, Switzerland). In the context of Type 1 diabetes, modeled in NOD mice, his group showed that certain microRNA levels (miRs-142-3p; -142-5p; -150; -155) are elevated in pancreatic beta cell islets, and in sorted beta cells, in the pre-diabetic state, and this is not in response to proinflammatory cytokines. However, these miR levels are increased in the infiltrating lymphocytes, begging the question that miRs may be transferred from leukocytes to beta cells. Jurkat cells (model CD4+ T cells) released EVs containing relatively high amounts of miRs-142-3p, -142-5p, and -155, which were indeed transferred to cultured beta cells upon incubation with the T cell EVs. These beta cells increased gene expression levels of several other chemokines in response to T cell EVs, and transfection of the beta cells with the miR mimics promoted the same expression changes. EV exposure also drove apoptotic beta cell death (without affecting other pancreatic cell types), and this was also mimicked by individual miR transfections into beta cells. While non-beta cells also take up lymphocyte EVs, only beta cells exhibited gene expression changes; of note, NF-kB nuclear translocation also occurred in the beta cells, suggesting a global driver of the cyto/chemokine expression changes. Gratifyingly, these effects were replicated in human tissues as well. Transfection of beta cells with anti-miRs apparently successfully prevented the impact of lymphocyte EV-transferred miRs, as this halted the apoptotic effect of the EVs. For an *in vivo* therapeutic attempt, the research group generated a “miR sponge” construct used in beta cell-targeted AAV transfection; this reduced the number of mice progressing with the disease. Continuing work will examine the roles of other non-coding RNAs that may be transferred from lymphocytes to beta cells.

Dennis Steindler (University of North Carolina; University of Florida, US) followed with more examples of exosome/microvesicle (EMV)-driven pathologies. He introduced the concept of adult neural stem cell-driven disease as a proliferative failure (e.g., Alzheimer’s and Parkinson’s disease) or a proliferative excess (e.g., brain tumors). Focusing on Parkinson’s disease, his group has identified human adult neural progenitor (AHNP) cells that are capable of proliferation and differentiate into neurons and glia. The cells are from cases of both idiopathic Parkinson’s and gene-identified Parkinson’s. These cells release EMVs that bear signature profiles in nanoparticle tracking analysis; in the case of gene-identified Parkinson’s, correction of the mutation returns the mutant EMV profile towards normal cell EMV profiles. This holds true for content of EMVs as well. There are interesting overlaps in neurodegenerative diseases between transmission of disease states from cell to cell with near infectious (viral-like) aspects. One hypothesis is that the neural connectome, perhaps via the vagus nerve, is hijacked to put such particles, including EMVs, into the central nervous system.

Moran Amit (MD Anderson Cancer Center, US) further connected the pathologic conditions (e.g., head and neck cancer) that alter normal cell function (neural reprogramming) via exosomes. Until recently, oncologists have viewed nerves involved in cancer as innocent bystanders. We now know that cancer cells can migrate or metastasize along neural tracks, and possibly have a more active role in tumor progression and malignancy. Tumor innervation is often associated with worse outcomes, but little is known about this. Using an *in vitro* ganglion + tumor cell assay, some tumor cells could promote neuritogenesis better than others; a common feature among those cells was the loss of p53, and this held true in murine studies, even in a pre-malignant stage prior to cancer cell/neuron contact. Thus, there should be a secreted component, which appears to be p53-deficient/mutant exosomes. miR-34a, present in p53wt exosomes, restricts neuron growth. In p53 mutant exosomes, in the absence of miR-34a, miR-21 and -324 strongly promote neuritogenesis. Phenotypically, the neurons are mostly sympathetic, have dysregulated pathways in stemness, proliferation, and neural transmission, and may even switch phenotypes. Beta-blockers may inhibit this neuronal-promoted tumor growth.

Tsuneya Ikezu (Boston University, US) told us about EV protein networks from iPSC-derived neural cells and brain tissue of patients with Alzheimer’s Disease (AD). High-level proteomic analyses of EVs from iPSC differentiated brain cell types and from brain tissues of patients with AD, mild cognitive impairment, and healthy controls led to specific markers for cell-type specific EVs and could clearly separate the EV types by protein composition. The statistical analyses could also distinguish EV protein subsets between healthy control brain EVs, patients with mild cognitive impairment, and AD patients, including some that might show progressive changes towards AD. Certain proteome modules resembled pathways found in activated astrocytes, including inflammatory processes. This information could form a basis for EV proteomics in liquid biopsy for AD development and monitoring.

Faisal Alibhai (University Health Network, Toronto, Canada) provided insight into a near universal human phenomenon, aging, and the associated changes in circulating EV cargo and function. Parabiosis of murine circulation involving young and old mice rejuvenates multiple organ systems in the old mouse, but the young mouse shows age related defects. Thus, blood circulating factors play roles in aging (and youth), begging the question of the roles of EVs in this. Faisal isolated EVs from young and old murine populations, noting that the particles from old individuals were both fewer and smaller than those from young plasma. Curiously, EV markers (CD63, CD81, TSG101) were more abundant in old plasma, while markers of lipoproteins were more abundant in young plasma, suggesting that the particle differences noted may be skewed in this fashion. In functional studies, peritoneal macrophages were treated with EVs from old and young plasma, and then were stimulated with LPS. Under both circumstances, old EVs decreased IL1B and IL12B expression (and other cytokines) more than young EVs. Old EVs also reduced VEGF-mediated endothelial cell tube formation. The miR content of old EVs targeted numerous pathways that could be dysregulated in aging (inflammation and senescence). In an attempt to reduce aging effects with senolytic therapy (dasatinib and quercetin in this case) in old mice, the treatments reduced miR levels in plasma EVs, and the EVs no longer blocked endothelial cell responses to VEGF. Thus, aging cells manifest senescent phenomena via aged EVs, and some of the effects may be reversed by senolytic therapies (or young EVs).

The last talk of the Pathology session was about the utility of neural stem cell EVs to improve outcomes in a porcine ischemic stroke model, presented by Steven Stice (University of Georgia, US). As mesenchymal stem cells (MSCs, and their EVs) are seeing use in many inflammatory-related neurologic diseases. Steve’s group found that neural stem cell (NSC) EVs better promoted an anti-inflammatory immune environment than MSC EVs in a murine stroke model. They chose a pig model as a better representative of human brains than rodent models. In this study, pigs were trained for motor and behavioral function; subjected to ischemic strokes; treated with NSC EVs; monitored by imaging and functional recovery studies; underwent further clinical testing and imaging; and followed finally, by an autopsy. Pigs treated with NSC EVs showed stunning functional recovery compared to surviving untreated pigs. On MRI assessment, the extent of midline shift - a pathology-induced mass effect leading to brain displacement off the midline - is correlated with survival. NSC EV-treated pigs showed reduced midline shift and increase in survival rate. On the pig-adapted Modified Rankin scale (mRS), pigs that received NSC EVs, irrespective of a high or low midline shift, showed reduced mRS scores, indicating less disability. Consistent with reduced inflammatory responses, pigs treated with NSC EVs demonstrated attenuated microglia activation both *in vitro* and *in vivo*.

The final session of Day 3 was devoted to EVs and viruses, and was hosted by Nihal Altan-Bonnet (NIH, National Heart, Lung, and Blood Institute, US) and Steve Gould (Johns Hopkins University, US). It started with James Erickson (George Mason University, US) discussing how to separate EVs from virions in Coronavirus infections. The lab has used several techniques to separate virus (typically HIV) from EVs in virally infected cells, including the use of differential ultracentrifugation with a high-resolution density (iodixanol) gradient ultracentrifugation. They applied this strategy to isolate a betacoronavirus (OC43, BSL2 compatible) from infected cells (lung cancer cell line). Incubating these virus fractions with Vero cells resulted in OC43 RNA transfer to the recipient cells. Also, viral RNAs were present in EV fractions. Upon noting that lymphopenia is associated with severe SARS-CoV-2 infections, they generated HEK transfectants expressing SARS-CoV-2 spike proteins or multiple viral proteins. EVs from these cells were incubated with T cells; EVs containing multiple viral proteins reduced cell viability, suggesting an EV-based mechanism for viral-induced lymphopenia.

Martin Olivier (McGill University, Canada) then presented the role of EVs where a virus infects another pathogen, in this case, the Leishmania RNA virus and the trypanosome Leishmania. The parasite can be transmitted through the bite of a sandfly, where it infects macrophages and neutrophils (and macrophage engulf the neutrophils).The parasite replicates as amastigotes in the macrophage, which can then transmit the amastigotes to another biting sandfly. Martin’s lab has studied how Leishmania hijack macrophage signaling and innate immune responses for propagation. These studies identified the pathogen metalloprotease GP63 as a virulence factor, but it was clustered in small entities in the macrophage. This led to the discovery of GP63 (and other virulence factors) in Leishmania exosomes/EVs, and the exosomes modulated macrophage signaling, inhibiting antimicrobial defenses and inducing inflammatory cell recruitment at the sites of infection (during a blood meal). Recently, a Leishmania RNA virus (LRV) was discovered that might be small enough to fit in Leishmania exosomes, raising the possibility that the virus could be transferred via exosomes. Indeed, the pathogen exosomes contained viral components that were detected biochemically, and electron microscopy showed the presence of the whole virus in about 30% of the exosomes. The virus adds to the skin hyperinflammation upon injection of exosomes, aiding and abetting the infection cycle, but potentially provides a new therapeutic target.

Hameeda Sultana (Old Dominion University, US) brought up another circumstance where host cell exosomes facilitate viral transmission. Zika virus (ZIKV) induces cell death in cortical neurons, with the infections in the differentiated and matured neurons. The infected neurons release exosomes containing viral RNA and proteins, and the exosomes can re-infect naïve cells. Hameeda’s group sought to understand the mechanisms behind the enhanced exosome release from infected cells that leads to further viral transmission. Neutral sphingomyelinase 2 (nSMase2; SMPD3), which is involved in numerous points of the uptake, in endosomal trafficking, and in vesicle release pathways, was found to be a logical player. In infected neurons, SMPD3 activity was higher in both the cells and their exosomes; silencing it reduced vesicle release and viral transmission. Thus, it appears that ZIKV induces SMPD3 activity, which leads to greater vesiculation, and thus increased passage of the virus.

In a setting where EVs may be used to actually mitigate viral disease, Heather Branscome (George Mason University) presented data on the use of stem cell EVs in the repair of cellular damage. The scenario involves HIV infection of neurospheres - a novel achievement in itself - as a model of CNS damage such as HIV-associated neurocognitive disorders (HAND), and the use of reparative stem cell EVs in healing cell damage. Stem cell therapies have been touted as a valuable means to treat various diseases, but a more potent activity may reside in stem cell EVs, which have reduced immunogenicity and a more facile handling and storage. She described the stem cell sources (mesenchymal stem cells, MSCs, and iPSCs) and described the tangential flow filtration (TFF) methodology for EV isolation. Stem cell (SC) EV cargo included cytokines (FGF2, VEGF, IL4) that differed between the two stem cell EV sources. The SC EVs could promote cell migration and endothelial cell tube formation, and the EVs were taken up by neurospheres with long half-lives (up to 9 days). In HIV-infected neurospheres, SC EVs reduced p53 (Ser-15) phosphorylation, suggesting a return to the cell cycle, and the EVs also reduced amounts of pro-inflammatory cytokines. Using isolated cell types (neurons, astrocytes, macrophage) exposed to ionizing radiation, SC EV treatment reduced apoptotic induction. The results suggest the ability of SC EVs to protect and repair cell damage even in the face of a brain HIV infection.

The final Virus session speaker was Daniel Pinto (Walter Reed Army Institute of Research, US) who told us of the role of EV in transmission and spread of the HTLV-1 virus. This virus can cause adult T cell leukemia/lymphoma, or a neurocognitive disorder known as HAM/TSP. A prevalent infectious motif is cell-cell contact. During EV preparations using density gradient centrifugation, they noted that viral components could end up in EV fractions that were separated from the virus itself, and from EVs containing virions. This begged the question, which of these are infectious? Curiously, none of them were separately infectious, nor were they infectious if all were combined. However, the treated cells displayed clustering/aggregation phenotypes similar to that seen in bona fide HTLV-1 infection. This perhaps enables the cell-cell contact that is necessary for viral spread. EVs from infected cells enhanced viral infection (measured by viral RNA load in recipient cells), and this could be blocked by antibodies against cell adhesion molecules CD45 and ICAM1 or by treating cells with siRNAs. In an animal model, injecting EVs prior to an infection with HTLV-1 increased the viral load in the blood and numerous target tissues of the mice. Differential centrifugation yielding 2K × g, 10K × g, and 100K × g fractions showed that most of the detrimental effects (and most of the viral components) were in the 2K and 10K fractions, along with differential cytokines (and surface *vs.* internal localizations). Thus, different EV subtypes may have unique effects on cells that ultimately lead to more efficient viral infection and spread.

**ASEMV2020 Day 4** started with the beneficial uses and therapeutic effects of EVs, hosted by Saumya Das (Massachusetts General Hospital, US) and Kendall van Keuren-Jensen (TGEN, US). Robert Blelloch (UCSF, US) opened up the session with his group’s studies showing how exosomes suppress anti-tumor immune responses. Unsurprisingly, this focused on the immune checkpoint inhibitor PDL1, part of the PD1/PDL1 checkpoint axis. T cell activation can result in expression of PD1 as a means of immune control; cells expressing its ligand PDL1 (often other immune cells, but also cancer cells) can suppress T cell activity as a control against autoimmunity. For instance, as activated T cells infiltrate tumors, tumor PDL1 could bind T cell PD1 to deactivate the T cell effector function, and this is the standard paradigm for use of immune checkpoint inhibitors. Robert’s lab believes there may be an important role of PDL1 far earlier in the priming phase of T cell activation. PDL1 is found on tumor-derived exosomes, but not all tumors may localize it there, suggesting that this may be an active process. Their additional data indicate that the vesicles are specifically exosomes and not other EV types. They employed a mouse prostate cancer model (TRAMP-C2) that is resistant to anti-PDL1 antibody (checkpoint inhibitor) therapy. However, the loss of PDL1 or reduced exosome formation (RAB27A or SMPD3 knockout) dramatically reduced tumorigenicity, implying PDL1 as critical to tumor progression; this also involved the immune system via T cell activation. Adding back PDL1-containing exosomes reversed the effects. This was particularly notable in draining lymph nodes, suggesting that T cell priming may be key. PDL1 on plasma exosomes is correlated with a lack of response to anti-PD1 therapy in patients across several cancer types. Their model invokes a tumor cell surface pool of PDL1 (more sensitive to checkpoint inhibition) and an exosome pool of PDL1, which impacts T cell priming in lymph nodes that remains resistant to such therapies.

Ryan Reshke (University of Ottawa, Canada) introduced a method for loading and delivery of siRNA in EVs. While siRNAs are quite effective, the delivery systems beyond liver targets (liver serves as a depot for lipoparticle delivery) can lead to systemic (and liver) toxicity, so better delivery means are needed. The number of small RNAs in EVs remains controversial, but appears to be limited, suggesting a need for efficient loading to improve therapeutic capacity. One novel concept involves the use of pre-miR-451, which is a uniquely short pre-miR hairpin that is too short for processing by DICER and is sliced by AGO2, and is highly enriched in EVs. The short hairpin is responsible for this lack of DICER processing and allows pre-miR-451 preferential EV loading. Other RNAs, such as siRNAs, can also be efficiently loaded, provided they have the miR-451 hairpin, allowing for their enrichment and packaging. Ryan’s group demonstrated this in a GFP silencing model, showing that in comparison to liposomal delivery, they could use 10-100-fold less siRNA to achieve silencing. In a translational setting of a murine model, they could knock down transthyretin (TTR) in the liver, where TTR mutations cause transthyretin amyloidosis. Their system used 100-fold less siRNA than a clinically-approved drug delivery particle. This is scalable (they have used this in non-human primates) and appears to be immunologically safe. They also showed utility in small intestine and kidneys, implying use for targets beyond the liver.

Lance Liotta (George Mason University, US) spoke of how to reverse tumor-induced immune suppression at the level of the sentinel lymph node. He initiated the discussion with a question: how do tumor EVs actually exit the solid mass of the tumor and traverse the extensive extracellular barriers to get into peripheral circulation? They believe that this “open door” is the lymphatic system for interstitial draining. This implies that tumor EVs in blood have likely already circulated through lymph nodes. Using tumor tissue directly, they are able to harvest EVs from the tumor interstitial fluid and separate them by 2K, 10K, and 100K × g fractions. They have also developed a nanoparticle (Nanotrap) that allows for controlled affinity release of cytokines that stimulates migration of immune cells (notably, antigen presenting cells) to remodel the lymph node. The accompanying goal is to have the appropriate tumor EVs (those with antigens for processing and presentation to T cells) enter the now-“hot” lymph nodes. These studies revealed that the 2K vesicles promoted both primary tumor growth as well as metastasis, while the 100K vesicles inhibited this tumorigenicity and progression. This may be related to VEGF content of the 2K fraction, as well as the autophagosome characteristics of that fraction; the roles of extracellular autophagosomes in cancer require further study.

Modification of EVs is another means of improving their delivery capability. Ikuhiko Nakase (Osaka Prefecture University, Japan) showed that one component of EVs as therapeutics is their loading with biofunctional molecules. The other component is targeting EV delivery. EV uptake is thus critical for cargo delivery, and internalization by endocytosis is considered a major pathway for this. However, Ikuhiko’s group found that active induction of macropinocytosis (via cancer-related receptors and oncogenic RAS) significantly enhances EV uptake. For instance, stimulation of EGFR enhances EV uptake by several orders of magnitude. Further, they created a modification of the Cathelicidin antimicrobial peptide (CAMP/CAP18), called sCAP18, that enhances cancer cell EV uptake by inducing macropinocytosis. They have also made sCAP12-2, and stearyl-modified versions of these for insertion into EV membranes. Interestingly, target-cell glycosaminoglycans were also required for the macropinocytotic uptake. Loading the EVs with saporin (ribosome inactivator) resulted in efficient cell killing.

In an eye-opening talk, James Patton (Vanderbilt University, US) discussed the roles of EVs in retinal regeneration. In retinal regeneration, the need is for quiescent stem cells to enter the cell cycle and proliferate. Knowing that EVs from KRAS mutant cancer cells can induce KRAS wild type cells to proliferate and invade, James’ group pursues EV-driven modes of retina regeneration. A cell type called Muller glia (MG) in the retina is responsible for generating all the retinal cell type upon damage. In zebrafish, this process is active in retinal regeneration; it is far less so for chicks, and even less active in mammals. Using the zebrafish model, they attempted to drive Muller glia proliferation by treating retinas with EVs from a KRAS mutant cell line (DKO1), which resulted in de-differentiation of some MG cells, along with some proliferation. These outcomes prompted a large-scale screen of 59 different types of EVs to measure proliferative responses in zebrafish eyes. They found that a rat glioma cell line, C6, provided active EVs. Further refinement of their EV isolation techniques identified a small EV/exosome fraction that carried the biologic activity. In attempts to boost the this activity, they found that overexpression of IL6 and ASCL1 in the C6 producer line did result in more active exosomes. Going forward, the research group hopes to produce designer exosomes capable of targeting MGs with high payloads of active cargo to promote retinal regeneration.

The final session of the conference was on EVs as biomarkers, conducted by hosts Julie Saugstad (Oregon Health and Science University, US) and Saumya Das (Harvard/MGH US). David Lyden (Weill Cornell Medicine, US) led the session with his group’s extensive work on extracellular vesicle and particle (EVP) cancer biomarkers. The Lyden lab discovered exomeres, non-membranous nanoparticles of ~30nm diameter, which are abundant at pre-metastatic niches; the small sizes of exomeres may contribute to their wide distribution capabilities. These EVPs are among a broad range of extracellularly-released materials possessing biologic content, including dsDNA, which their group found mostly on EV surfaces. Using various separation techniques, including AF4 following differential ultracentrifugation, they categorize EVs into Exo-L (90-120 nm), Exo-S (60-80 nm), and exomeres (< 50 nm), cognizant that there are also larger vesicles. The diversity of the vesicles may relate to the diversity of local and systemic consequences of cancer. The biodistribution of cancer EVs *in vivo* likely relates to surface molecules that interact with other cell surface markers at metastatic organ sites. Their work implies that the organotropism of metastatic tumors may be initiated by the tumor EVs, particularly relating to EV integrin content (which may differ in ratio to the parental cell content). The overall impact of EV-driven cancer biology led them to analyze the proteomes of EVs from > 400 human cancer samples (cell lines, tissue explants and numerous biofluids). The goals were to define common human exosome markers from different source types (tissues, cell lines and biofluids); to identify tumor-specific exosome biomarkers; and to classify primary tumors of unknown origins by exosome proteome signatures. Some notable proteins include HSPA8 and CD9 present in/on many cancer exosomes, but CD63 served mostly as a marker for murine exosomes. The group has many matched samples of tumors, adjacent (and sometimes distant) normal tissues, and plasma, which allow for tissue explant exosome comparisons intra-patient. The proteome profiles may represent biomarkers and therapeutic targets, and the normal tissue serves as a means against drug targets that would affect normal tissue. Pathway analysis derived from pancreatic cancer exosome proteomes found epithelial-mesenchymal transition (EMT), coagulation, and TGFβ signaling as highest ranking. For lung cancer, the pathways were EF2, G2M checkpoint, and MYC targets. Across the 18 cancers in their study, certain proteins stood out as pan-cancer markers such as THBS2, VCAN, SRRT, and TNC. For pancreatic cancer, they identified 50 exosome proteins from patients that were not present in age/sex matched controls. Across cancers, plasma exosomes had sets of cancer proteins, but also sets of immune proteins that were not found in healthy donors.

A new area for EV studies, EVs in cancer related fatigue (CRF), was introduced by Dilorom Sass (NIH, National Institute of Child Health and Human Development, US). In CRF, there is an unusual sense of physical, emotional, or cognitive tiredness related to cancer or cancer treatment. CRF affects 30%-90% of cancer patients receiving therapy, and 30% will continue to experience such fatigue, months or even years post-treatment. Prior work has suggested that a wide variety of cytokines may be involved. Circulating EVs bearing cytokines could be systemic mediators and biomarkers of such inflammation. Dilorom’s study searched for associations between levels of fatigue in men after radiation treatment for prostate cancer, determined by questionnaire, and 45 plasma EV-associated immune markers. Hierarchical clustering of the cytokine readouts identified two clusters. A closer analysis found that eotaxin, HSP27, IL3, IP-10, and MIP3 alpha were significantly higher in the EV samples from the fatigued cohort. These studies raise questions in terms of measurements of plasma cytokine values: what fraction is actually measured, and are we overlooking a valuable source of potentially protected cytokines associated with EVs?

Janusz Rak (McGill University, Canada) introduced “leukobiopsy” as a variant of our typical takes on liquid biopsy. The successes of current liquid biopsy, biofluid-based biomarker information relevant to disease states, are significant in some areas, far less so in others. Janusz’s group had demonstrated transfer of the oncogenic EGFR variant, EGFRvIII (found in gliomas), from aggressive cancer cells that expressed the protein to more indolent cells that did not express it. This converted the indolent cells into far more aggressive tumors; the presence of EGFRvIII on vesicles in blood suggested that it could be a cancer-specific biomarker. As they extended studies into the RAS oncogene, they noted that cells receiving EVs from RAS mutant cells now possessed RAS protein, RNA, and DNA, implying an extraordinary passage/delivery capacity. Using various assays, the group demonstrated that normal cells could also take up cancer vesicles, and in certain cases, with observable effects (transient growth in soft agar, presence of micronuclei). From the perspective of the cancer cell, vesiculation and extracellular release of materials is programmed and is a necessary part of the cancer cell’s existence. There is another side, however: recipient cells who bind and take up cancer cell EVs, and process the contents. Janusz’s lab pursued this in the form of mutant RAS DNA in the blood components of tumor-bearing mice. They were able to find circulating RAS DNA, cell-free and in EVs, but the most abundant depot of the DNA was in white blood cells. Neutrophils in particular seemed to control the amounts of tumor DNA in blood; if those cells are eliminated, circulating tumor DNA and DNA in EVs increased. These data suggest a possible new form of liquid biopsy - leukobiopsy, where leukocytes may harbor the informative characteristics of circulating tumor materials and probed for those tumor entities, be they DNA, RNA, oncoproteins, or oncometabolites.

As introduced earlier by NIH/NIDA’s John Satterlee, there is a genuine need for biomarkers for drugs of addiction and substance use disorders. Here, Ursula S. Sandau (Oregon Health and Science University, US) described her work in EVs regarding methamphetamine use and treatment monitoring with EV cargo as a metric for recovery. Methamphetamine use is increasing globally, and can have damaging effects across organ systems, including adverse neuropsychiatric effects. Methamphetamine acts at synaptic dopamine transporters to block dopamine re-uptake, leaving dopamine in the dopaminergic neuronal synapse and stimulating a reward response. The drug also drives neuroinflammatory responses and has multiple implications. Brain microRNAs are altered in response to methamphetamine dosing, and these in turn, can affect proteins implicated in addiction; some of these miRs become diminished in plasma. Certain miRs are also implicated in blood-brain barrier permeability, allowing vesicle release into the blood compartment. In a collaborative study, Ursula’s group aimed to characterize plasma EVs in active methamphetamine users, and identify miRs with altered expression in that population. Using clinical data gathered as part of the study, the goal was to follow plasma EV miR changes in the context of clinical and neuropsychological changes in users.They performed vesicle flow cytometry to calculate particle concentrations in the subjects’ plasma, and found finding that measures of lifetime exposure showed some correlation to particle quantity. These correlations were maintained when sorting for EVs *vs.* all particles. Purification of EVs and quantification of miRs showed that 20 miRs were significantly increased, and 69 miRs decreased. Following statistical management, they narrowed the numerically relevant miR numbers to six. When correlating these to subjects’ clinical features, characteristics of lifetime exposure were significant and included frequency for three of the six miRs. Pathway analysis based on miR targets revealed pathway involved in cardiac, liver, and kidney disease, as well as neurological function. These also correlated with behavioral function related to methamphetamine use. The results of the study suggest that plasma EVs and their miR content may serve as biomarkers in methamphetamine use disorder and may correlate with clinical features.

The final speaker of the session, and of the conference, was Andy Hill (LaTrobe University, Australia), regarding EV-based biomarkers of neurodegenerative disease. Neurodegenerative diseases present with a range of signs and symptoms, and with clinical decline before diagnosis. Causes are also variable, but often involve protein misfolding and deposit in the brain. As diagnosis may involve sophisticated brain imaging techniques not routinely available, the search for blood-based biomarkers is reasonable. EV microRNAs have been sought as potential biomarkers for years, particularly in accessible, minimally-invasive sources, such as blood. However, blood-based biomarkers might not represent the actual pathology of the brain, so Andy’s group delved into techniques to isolate vesicles from actual brain tissues (both healthy controls and AD patients). The EV cargo was then compared to the blood (serum) EV cargo they had previously identified as putative AD biomarkers. There were indeed miRs that were found at higher levels in AD brain EVs *vs.* control brain EVs, but also some that were different between serum and brain. Thus, the serum EV miRs were not exactly the same as the brain EV miRs, but could nonetheless be useful. The next step was to compare the miRs predictive of AD with imaging studies. Employing machine learning (Association Rule Mining, ARM), EV miR levels could predict imaging positive (AD) and imaging negative (healthy control) cases. The brain-serum EV work was extended to Parkinson’s Disease (PD), where they found miRs that could distinguish PD stages. They further extended the brain EV work to amyloid lateral sclerosis (ALS) patients, where they identified potential protein and miR biomarkers that relate to ALS pathology. Some of their early work was done in prion disease in mice, which eventually led to brain and blood sampling to identify EV biomarkers that could be used for Creutzfeldt-Jakob disease (CJD) in humans. This work represents a tour de force of investigating disease characteristics of neuropathologies and their relationships to potential blood-based biomarkers.

In the last two portions of the meeting, Fatah Kashanchi (George Mason University, US) held an interactive workshop on grant writing aimed especially at obtaining funding via the US NIH aimed at young (and not so young) investigators. He supplied potential applicants with the perspective of a grant reviewer, and what the reviewers want and expect to see, and he incorporated NIDA’s John Satterlee’s “ten commandments for preparing a compelling R01 application”. He then provided an example of a Specific Aims page and gave rationales for what was stated, why reviewers need and want specific information, and how the applicant should supply that.

The Closing Ceremonies also served as an award platform for young investigators; these included speakers:

Moran Amit (MD Anderson, US)
Frederik Verweij (Institute of Psychiatry and Neuroscience of Paris, France)
Hannah McMillan (Duke University, US)

Poster awards went to

Killian O’Brien (Harvard/MGH, US)
Kathleen Lennon (City of Hope, US)

As the ASEMV and the exosome/EV field have grown over the years, the areas of biology and pathology studied have increased as well. While much research has focused on cancer in the “early years”, we see in this conference the breadth of diseases studied, including the basic biology of vesicles in normal tissues, systems, and organisms. Vesicles are now viewed as communicators between and among cells, as well as between hosts, pathogens, symbionts, and kingdoms. The analytical and separation technologies have advanced, while some of the classical techniques continue to maintain their value. Every year brings startling changes, more information, and often more confusion, which one might describe as science in its element.

The ASEMV Annual Meeting for 2021 is already in the planning phase and we hope to meet again in person, and continue to highlight some of the best of exosome/extracellular vesicle science.

ASEMV would like to thank the Organizing Committee for their efforts, and in particular, Roger Alexander for his tireless work in preparing the online format.

Louise Laurent, UC San Diego, ASEMV2020 Organizing Committee Chair

Steve Gould, Johns Hopkins University, ASEMV President

Roger Alexander, Extracellular RNA Communication Consortium

Nihal Altan-Bonnet, NIH NHLBI

Michael Graner, University of Colorado Anschutz

Kendall Jensen, Translational Genomics Research Institute

Fatah Kashanchi, George Mason University

Meta Kuehn, Duke University

Leonid Margolis, NIH NICHD

Janusz Rak, McGill University

Matt Roth, Baylor College of Medicine

Julie Saugstad, Oregon Health & Science University

## Declarations

### Copyright

©The Author 2020.

